# Crimean-Congo Hemorrhagic Fever with Acute Subdural Hematoma, Mauritania, 2012

**DOI:** 10.3201/eid2207.151782

**Published:** 2016-07

**Authors:** Ahmed S. Kleib, Sidi M. Salihy, Sidi M. Ghaber, Baba W. Sidiel, Khalil C. Sidiya, Ely S. Bettar

**Affiliations:** Centre Hospitalier National, Nouakchott, Mauritania (A.S. Kleib, S.M. Salihy, S.M. Ghaber, B.W. Sidiel, K.C. Sidiya, E.S. Bettar);; Université des Sciences, de Technologie et de Médecine, Nouakchott (A.S. Kleib, S.M. Salihy, S.M. Ghaber, B.W. Sidiel, E.S. Bettar)

**Keywords:** Crimean-Congo hemorrhagic fever, CCHF, Crimean-Congo hemorrhagic fever virus, viruses, Mauritania, intracranial hematoma, acute subdural hematoma

**To the Editor:** Crimean-Congo hemorrhagic fever (CCHF) was first described in Crimea in 1944 and in the Congo in 1969. Since then, many cases in humans have been reported from different regions (*1*–*3*). The disease is transmitted to humans through the bite of an infected tick or by direct contact with blood or tissue from infected humans and livestock. We report an unusual case of acute subdural hematoma secondary to CCHF.

A 58-year-old man, a shepherd, was admitted to Centre Hospitalier National (Nouakchott, Mauritania) on July 2, 2012, with fever and epistaxis. One week earlier, he had fever, nausea, and vomiting. Without biologic confirmation of the infection, his doctors treated him for malaria. His leukocyte count was 3,200 cells/mm^3^ (reference range [RR] 4,000–10,000 cells/mm^3^), hemoglobin level was 10.6 g/dL (RR 14.0–17.5 g/dL), and platelet count was 22,000/mm^3^ (RR 200,000–400,000 cells/mm^3^). His aspartate aminotransferase level was elevated to 162 IU/L (RR 8–30 IU/L), and his alanine aminotransferase level was elevated to 200 IU/L (RR 8–35 IU/L). Glasgow Coma Scale score was 15. Results were positive from tests for CCHF virus-specific IgM by ELISA and CCHF virus by real-time reverse transcription PCR.

Treatment with platelet transfusions and supportive therapy was initiated. Fever and epistaxis improved on the third day of admission. On hospitalization day 6, headache and acute encephalopathy developed in the patient. Glasgow Coma Scale score was 13 (Figure, panel A). A computed tomography (CT) scan of his head without contrast showed acute subdural hematoma on the left side. On day 16 of admission, the patient’s general condition worsened; he became more obtunded (experienced reduced consciousness), and right-sided upper limb hemiparesis developed. A repeat CT scan of his head showed a subdural hematoma with surrounding edema and midline shift (Figure, panel B).

Our care team considered a conservative management approach. We gave the patient corticosteroids and saline. After 4 weeks, his symptoms had improved markedly and he was discharged in stable condition. A 1-month follow-up CT scan of his head without contrast showed complete resolution of the subdural hematoma (Figure, panel C). Thrombocytopenia could be considered a risk factor for the development of a spontaneous acute subdural hematoma of arterial origin with more rapid and aggressive evolution (*4*).

The main vector for CCHF virus transmission appears to be ticks from the genus *Hyalomma* (*2*). CCHF that affects multiple organs is characterized by fever, myalgia, headache, shock, disseminated intravascular coagulation, recurrent extensive bleeding, and thrombocytopenia. After 5–6 days of illness, petechial rash, signs of bleeding (e.g., hematemesis and melena), and liver failure occur. CCHF can be diagnosed by using serologic tests to detect IgM and IgG against the virus and by using molecular-based techniques, such as conventional and real-time reverse transcription PCRs, to detect the genome of the virus (*5*,*6*).

Brain hemorrhage in persons with CCHF is rare. We report a case of acute subdural hematoma secondary to CCHF, where thrombocytopenia was the main cause of cerebral hemorrhage. Management of this case was challenging due to the underlying bleeding tendency of the patient and risk for nosocomial infection. We provided conservative treatment and the patient showed total remission. The patient improved due to the use of corticosteroids and the natural progressive resorption of blood.

Alavi-Naini et al. reported a case of CCHF in a person with a bilateral frontal parasagittal hematoma that was managed with oral ribavirin and intravenous ceftriaxone, platelet transfusions, and supportive therapy (*5*). The patient recovered. Kumar et al. reported 5 case-patients with dengue hemorrhagic fever and intracranial bleeding. Two of these patients underwent surgery after platelet transfusion and recovered (*7*). A high case-fatality rate has been reported in many countries among persons who became infected with CCHF after having contact with a hospitalized CCHF patient (*2*). Swanepoel et al. reported a case of CCHF in which the patient died of complications following surgical intervention for cerebral hemorrhage (*8*).

Death from CCHF usually occurs after 5–14 days of illness (*1*,*8*,*9*). The basic pathogenesis of CCHF virus at the molecular level is complex and not well defined. Endothelial cells, immune response, virus load, and coagulation cascade play major roles in the disease pathogenesis. Blood and endothelium appear to be the target tissues of the disease (*9*). The coagulation cascade becomes activated over 24–48 hours; however, thrombin becomes activated and promotes edema formation and further disruption of the integrity of the blood–brain barrier. The edema formation starts when erythrocytes in the hematoma begin to lyse and its degradation products are deposited into the brain parenchyma, initiating a potent inflammatory reaction (*10*).

Although surgery remains the first choice for the treatment of acute subdural hematoma, some patients may benefit from conservative management with careful monitoring. This report highlights the value of an early diagnosis of CCHF and neuroimaging for severe cases when brain hemorrhage is suspected.

**Figure:**
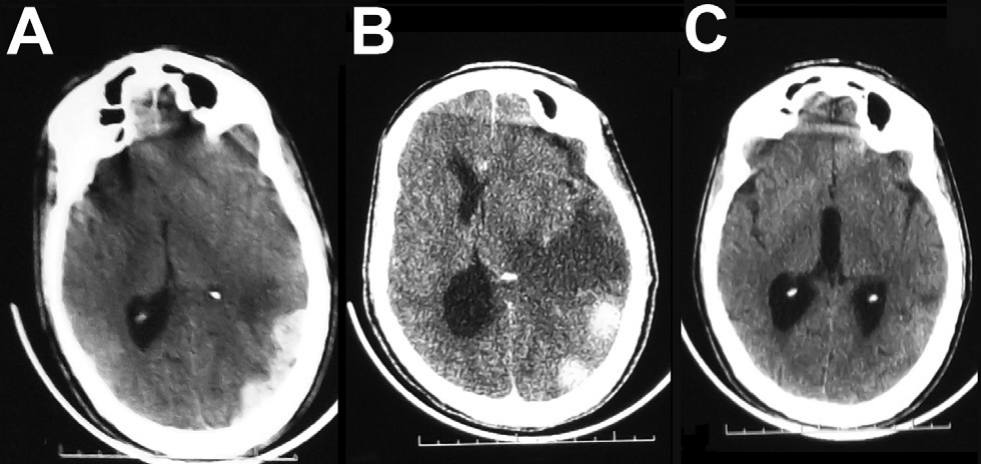
Computed tomography scan image of the brain of a 58-year-old man with Crimean-Congo hemorrhagic fever, Mauritania, 2012. A) Acute subdural hematoma, on the left side. B) Subdural hematoma with perihematomal edema and midline shift. C) Complete resorption of the subdural hematoma with residual edema, 1 month later.
